# Preparation of NH_2_-MIL-101(Fe) Metal Organic Framework and Its Performance in Adsorbing and Removing Tetracycline

**DOI:** 10.3390/ijms25189855

**Published:** 2024-09-12

**Authors:** Yiting Luo, Rongkui Su

**Affiliations:** 1 School of Business, Hunan First Normal University, Changsha 410114, China; 2 National Engineering Laboratory of Southern Forestry Ecological Application Technology, Changsha 410004, China; 3 College of Life and Environmental Sciences, Central South University of Forestry and Technology, Changsha 410004, China

**Keywords:** metal–organic framework materials, NH_2_-MIL-101(Fe), tetracycline, adsorption, kinetic analysis, adsorption thermodynamics

## Abstract

Tetracycline’s accumulation in the environment poses threats to human health and the ecological balance, necessitating efficient and rapid removal methods. Novel porous metal–organic framework (MOF) materials have garnered significant attention in academia due to their distinctive characteristics. This paper focuses on studying the adsorption and removal performance of amino-modified MIL-101(Fe) materials towards tetracycline, along with their adsorption mechanisms. The main research objectives and conclusions are as follows: (1) NH_2_-MIL-101(Fe) MOF materials were successfully synthesized via the solvothermal method, confirmed through various characterization techniques including XRD, FT-IR, SEM, EDS, XPS, BET, and TGA. (2) NH_2_-MIL-101(Fe) exhibited a 40% enhancement in tetracycline adsorption performance compared to MIL-101(Fe), primarily through chemical adsorption following pseudo-second-order kinetics. The adsorption process conformed well to Freundlich isotherm models, indicating multilayer and heterogeneous adsorption characteristics. Thermodynamic analysis revealed the adsorption process as a spontaneous endothermic reaction. (3) An increased adsorbent dosage and temperature correspondingly improved NH_2_-MIL-101(Fe)’s adsorption efficiency, with optimal performance observed under neutral pH conditions. These findings provide new strategies for the effective removal of tetracycline from the environment, thus holding significant implications for environmental protection.

## 1. Introduction

Tetracycline is a broad-spectrum, widely used antibiotic known for its low cost and extensive application. It ranks as the world’s second-largest antibiotic with annual production reaching several thousand tons [1]. When tetracycline is ingested by organisms, over 75% of its parent compound or metabolites are excreted via urine and feces, ultimately entering aquatic environments [2]. Featuring a stable tetracyclic structure, its stability and high hydrophilicity facilitate accumulation in water, where it resists degradation and bioaccumulates through aquatic organisms into the food chain [3,4], thereby impacting human health. Moreover, high concentrations of tetracycline in water environments exert toxic effects, posing potential threats to aquatic ecosystem communities [2,5]. Additionally, tetracycline in soils can enter water environments via runoff, and its abundant presence can induce genetic mutations in bacteria, leading to antibiotic resistance, thereby posing greater risks to human health [6]. Consequently, the urgent need to remove such biologically persistent pollutants from wastewater is imperative [7].

Currently, the methods for addressing tetracycline pollutants in water can be broadly categorized into three main classes: biological, chemical, and physical approaches [8,9,10]. Biological degradation processes find extensive application in wastewater treatment, relying on microbial metabolic activities to progressively convert large molecular compounds into smaller ones [11,12,13,14]. However, these degradation methods often require prolonged durations and encounter challenges such as selecting and domesticating antibiotic-resistant microorganisms. Furthermore, the inherent biotoxicity of antibiotic molecules and their relatively low bioavailability can adversely affect the overall efficacy of biological degradation technologies, potentially inhibiting or completely nullifying their effectiveness in certain scenarios [14]. Chemical methods for tetracycline pollution treatment primarily involve oxidative decomposition pathways, converting large-molecular tetracyclines into smaller molecules to reduce environmental hazards. Currently, advanced oxidation processes (AOPs) are widely used chemical methods [15,16,17]. Although these technologies boast high treatment efficiencies, they may pose drawbacks such as secondary pollution and a limited cost-effectiveness. Moreover, residual chemical toxicity from the small-molecule substances generated after treatment is also a concern [18,19]. Physical methods predominantly employ adsorption, membrane separation technologies, or solvent extraction to remove tetracyclines from water [20], with adsorption being particularly prevalent due to its simplicity, flexibility, and cost-effectiveness [21,22]. The adsorption process does not generate harmful substances, making it widely regarded as an efficient and extensively applicable method for removing tetracyclines from water bodies [23]. In summary, developing stable, efficient, and environmentally friendly materials for tetracycline adsorption not only holds profound practical value but also carries significant economic potential [24].

Metal–organic frameworks (MOFs), as a class of inorganic–organic composite porous materials, are emerging as a frontier in materials science research [25,26,27,28]. MOFs have garnered attention for their exceptionally high surface area, structural stability in liquid phases, rigid frameworks resistant to deformation, and abundant unsaturated metal sites, making them highly promising adsorbents. Traditional MOFs can be categorized into several types, including the UiO series (Universitetet I Oslo), ZIF series (Zeoliticimidazolate Frameworks), and MIL series (Materials Institute Lavoisier Frameworks) [29]. Among them, a series of materials of Institute Lavoisier frameworks (MILs) reported by the French Férey research group have been the most widely studied materials over the years [30,31]. The main metals of the materials are aluminum, chromium, iron, indium, vanadium, gallium, and other salt compounds, and the organic ligands are terephthalic acid and pyromellitic acid (1,3,5-H_3_BTC). At present, MIL-47, MIL-53, MIL-88, MIL-100, and MIL-101 have been successfully reported. MIL-101 is one of the representatives with good chemical stability and a large specific surface area and pore cage size, so it is applied in various fields. Zhang et al. used MIL-101-Cr to adsorb and remove the anionic dyes Congo red and methyl orange in wastewater, showing excellent adsorption performance [32]. The adsorption capacity was up to 1000 mg/g, indicating the great potential of the material in the treatment of dyes in wastewater. Synthesis methods for MOFs primarily include solvothermal synthesis, sonochemical synthesis, microwave-assisted synthesis, and mechanochemical synthesis [33,34]. Among these, solvothermal synthesis stands out as the most widely used and classic method for MOF synthesis. Its principle is akin to the natural formation process of minerals, involving a thermal reaction in specific solvent environments to create materials with tailored structures and properties [35]. Functionalized MOFs modified with various functional groups exhibit unique properties [36]. Currently, research on MOF modification is expanding, focusing on both pre-synthetic ligand modification [37,38] and post-synthetic host material modification [39]. The former involves predesigned modifications of ligands before material synthesis, while the latter enhances their functionalities after material formation. Amino functionalization is a commonly employed method for MOF modification, enhancing their adsorption performance, stability, wetting and oleophobicity improvement, adhesion enhancement, and tunable surface properties, thereby offering excellent performance and extensive applicability across various applications [40].

This study developed an amino-functionalized MIL-101(Fe) material, NH_2_-MIL-101(Fe), for the efficient adsorption and removal of residual tetracycline from water. The research focused on the following key aspects: (1) the development of a modification method for iron-based MOF materials, successfully synthesizing amino-functionalized MIL-101(Fe) material; (2) evaluating the performance and mechanism of NH_2_-MIL-101 (Fe) adsorption for tetracycline removal; and (3) an investigation of the effects of different adsorbent dosages, temperatures, and pH values on the adsorption and removal of tetracycline by the NH_2_-MIL-101 material.

## 2. Results and Discussion

### 2.1. Characterization of NH_2_-MIL-101(Fe)

#### 2.1.1. X-ray Diffraction Analysis (XRD)

**Figure 1:**
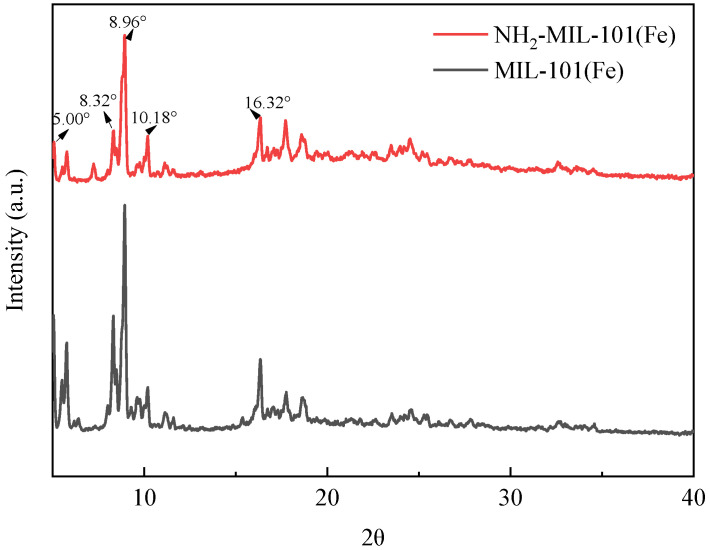
XRD spectrum of MIL-101(Fe) and NH_2_-MIL-101(Fe).

Figure 1 depicts the X-ray diffraction (XRD) pattern of MIL-101(Fe) and NH_2_-MIL-101(Fe). Clear characteristic peaks are observed at 5°, 8.32°, 8.96°, 10.18°, and 16.32°, corresponding to the specific crystallographic planes of NH_2_-MIL-101(Fe). The high quality of crystalline growth indicates excellent crystallinity, consistent with prior literature reports, thereby confirming the successful synthesis of NH_2_-MIL-101(Fe) [41,42]. In addition, the main diffraction peaks of NH_2_-MIL-101(Fe) are similar to those of MIL-101(Fe), indicating that the crystal structure is well preserved after the introduction of amino groups in MIL-101(Fe) [43].

#### 2.1.2. Fourier-Transform Infrared Spectroscopy (FT-IR)

**Figure 2:**
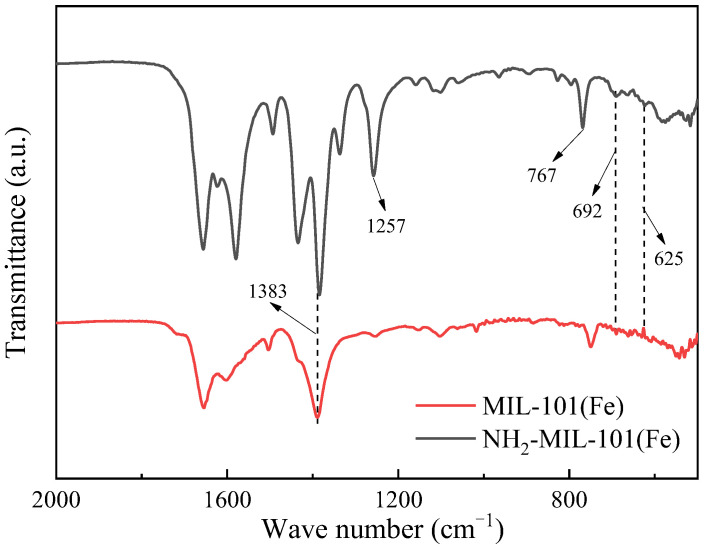
FT-IR spectrum of NH_2_-MIL-101(Fe) and MIL-101(Fe).

The FT-IR spectrum of MIL-101(Fe) and NH_2_-MIL-101(Fe) is depicted in Figure 2. In this spectrum, two peaks located at 625 cm^−1^ and 692 cm^−1^ can be observed, representing Fe-O bonds [42]. Meanwhile, a peak corresponding to the -COO^−^ vibration was observed at 1383 cm^−1^, confirming the presence of dicarboxylic acid linkers in NH_2_-MIL-101(Fe) and MIL-101(Fe) [44]. The peaks located at approximately 767 cm^−1^ and 1257 cm^−1^ are attributed to N-H and C-N bonds, respectively, indicating that the amino group has been successfully grafted onto NH_2_-MIL-101(Fe) [45]. In summary, the analysis of the infrared spectrum convincingly demonstrates the successful synthesis of NH_2_-MIL-101(Fe).

#### 2.1.3. Scanning Electron Microscopy and Energy-Dispersive Spectroscopy (EDS)

**Figure 3:**
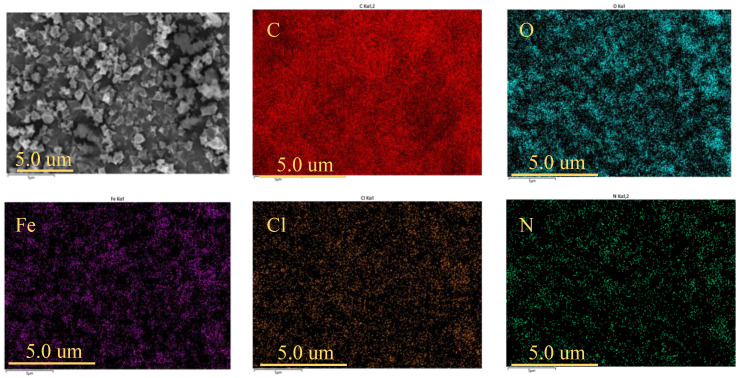
SEM and EDS images of NH_2_-MIL-101(Fe).

Scanning electron microscopy allows for the visual observation of the surface characteristics and microstructures of samples [3]. As shown in Figure 3, pure-phase NH_2_-MIL-101(Fe) exhibits a stable crystalline morphology with a uniform size, smooth surface, and a typical octahedral structure, ranging in width from 200 to 800 nm, consistent with the previous literature [46,47]. The infrared spectrum of the sample indicates the successful synthesis of NH_2_-MIL-101(Fe). To gain deeper insights into the composition and structure of NH_2_-MIL-101(Fe), further EDS spectroscopic testing was conducted. The test results clearly show the uniform distribution of the major elements C, O, N, and Fe in the NH_2_-MIL-101(Fe) sample, indicating that FeCl_3_ and NH_2_H_2_BDC successfully underwent coordination reaction in the DMF (N,N-dimethylformamide) solvent to produce the targeted product, NH_2_-MIL-101(Fe). Comparing the SEM images of NH_2_-MIL-101(Fe) (Figure 3) and MIL-101(Fe) (Figure S2), it is evident that NH_2_-MIL-101(Fe) maintains its morphology unchanged after the introduction of amino groups, indicating that amino decoration has no effect on the crystal morphology [43].

#### 2.1.4. X-ray Photoelectron Spectroscopy (XPS)

**Figure 4:**
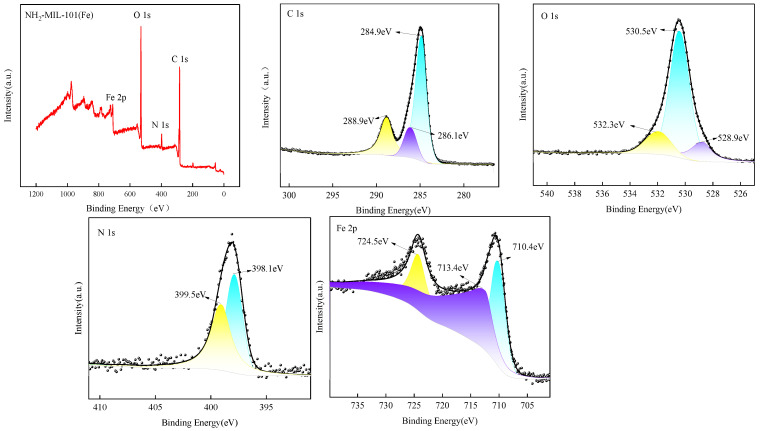
XPS spectrum of NH_2_-MIL-101(Fe).

X-ray photoelectron spectroscopy (XPS) testing yielded the full spectrum and orbital spectra of NH_2_-MIL-101(Fe), as depicted in Figure 4, confirming the presence of C, N, O, and Fe elements as expected. In the C 1s spectrum, the peak at 284.9 eV represents an overlap of C-C, C=C, and C-H bonds, typically associated with carbon-based materials, while the peak at 286.1 eV corresponds to the C-N bond. The peak at 288.9 eV is attributed to the COO- feature, potentially due to surface oxidation or the adsorption of carboxyl-containing impurities. In the O 1s spectrum, the peak at 532.3 eV is commonly associated with adsorbed oxygen on catalyst surfaces, likely due to exposure of the material to air, while the peak at 530.5 eV corresponds to oxygen in the 2-amino terephthalic acid ligand within the NH_2_-MIL-101(Fe) organic ligands, and the peak at 528.9 eV is characteristic of the Fe-O bond, further confirming a successful coordination between Fe ions and organic ligands. In the N 1s spectrum, the peak at 398.1 eV corresponds to the -NH_2_ group, a component of NH_2_-MIL-101(Fe) organic ligands, and the peak at 399.5 eV corresponds to N-C [48]. In the Fe 2p spectrum, the peaks at 710.4 eV and 724.5 eV correspond to Fe 2p 3/2 and Fe 2p 1/2, typical of Fe^3^⁺, with the peak at 713.4 eV being the satellite peak of Fe 2p 3/2, a distinct marker of Fe^3^⁺, further confirming the presence of Fe^3^⁺ in the sample [49].

#### 2.1.5. Specific Surface Area and Pore Size Analysis (BET)

**Figure 5:**
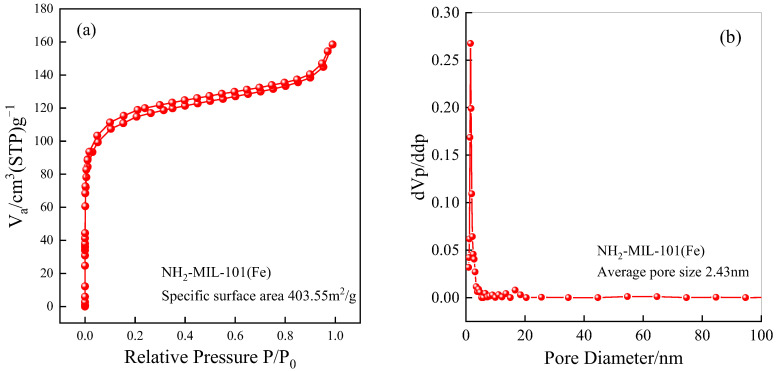
N_2_ adsorption–desorption isotherm (**a**) and pore size analysis (**b**) of NH_2_-MIL-101(Fe).

The BET model was used to calculate the specific surface area, and the pore size was calculated by the Barrett–Joyner–Halenda (BJH) model. As depicted in Figure 5, the N_2_ adsorption–desorption isotherms of the synthesized material were observed. The isotherm conforms well to Type IV as defined by the International Union of Pure and Applied Chemistry (IUPAC), indicating the material’s characteristic mesoporous nature. A rapid increase is distinctly observed in the low relative pressure region of the curve, highlighting the microporous nature of the material. Additionally, a nearly horizontal H4-type hysteresis loop is evident in the plot, indicating NH_2_-MIL-101(Fe) possesses a composite porous structure combining microporous and mesoporous characteristics. The pronounced filling effect in the low-pressure region is attributed to the initial gas adsorption in micropores, characterized by narrow pore channels. These slit-like features not only underscore its unique structural advantages but also provide favorable conditions for specific adsorption and catalytic processes. This hybrid micro-mesoporous structure enables NH_2_-MIL-101(Fe) to exhibit a notable performance across various applications.

Further pore size analysis was conducted to explore the material’s pore characteristics, as shown in Figure 5. The pore size distribution of the material primarily centers around the microporous region of 2 nm, with a main peak at 1.67 nm, indicating a narrow distribution. However, it also encompasses a broader range of mesopores, suggesting the presence of both micropores and mesopores within the material. Larger pore sizes may originate from internal cavities within particle aggregates or voids formed between stacked particles.

A comparison of specific surface area and average pore diameter data between MIL-101(Fe) and NH_2_-MIL-101(Fe) is presented in Table 1. NH_2_-MIL-101(Fe) exhibits a significantly reduced specific surface area compared to MIL-101(Fe), with slightly smaller pore diameters. This reduction is likely due to the introduction of amino groups, which occupy some of the surface and pore space of NH_2_-MIL-101(Fe). Through ammonia functionalization, ammonia molecules are directed to the center of the microporous cage, which makes nitrogen diffusion difficult and leads to the reduction of the partial adsorption space on NH_2_-MIL-101(Fe) [50].

#### 2.1.6. Thermogravimetric Analysis (TGA)

**Figure 6:**
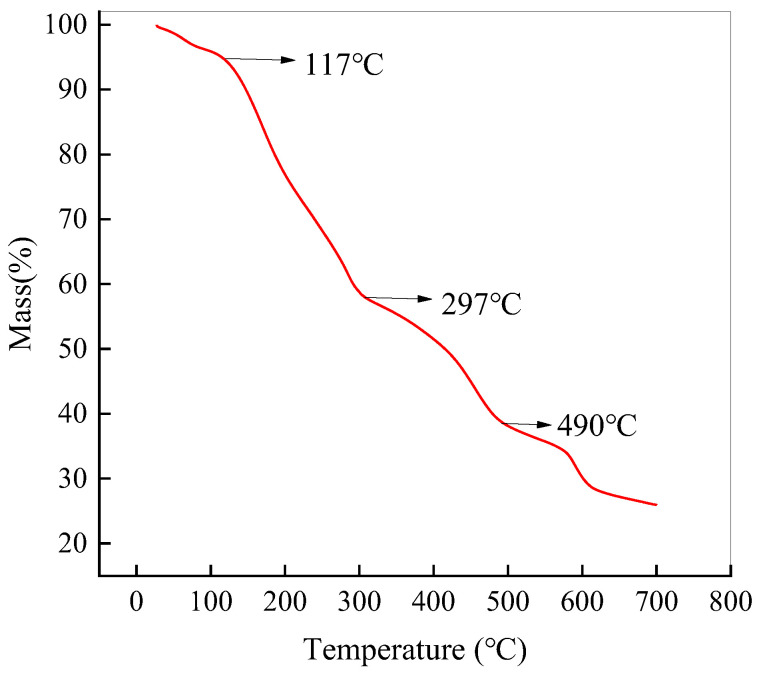
Thermogravimetric analysis of NH_2_-MIL-101(Fe).

Figure 6 illustrates the thermogravimetric analysis curve of NH_2_-MIL-101(Fe), which can be broadly divided into four stages of thermal weight loss. Below 117 °C, the predominant process involves the evaporation of adsorbed water and organic solvents. From 117 to 297 °C, volatile organic solvent molecules within the pores and reactants that have not fully participated in reactions begin to volatilize. Between 297 °C and 490 °C, the coordination bonds within NH_2_-MIL-101(Fe) start to break [51]. Beyond 490 °C, the metal–organic framework experiences complete collapse. Therefore, NH_2_-MIL-101(Fe) exhibits favorable thermal stability.

### 2.2. Adsorption Kinetics Analysis

**Figure 7:**
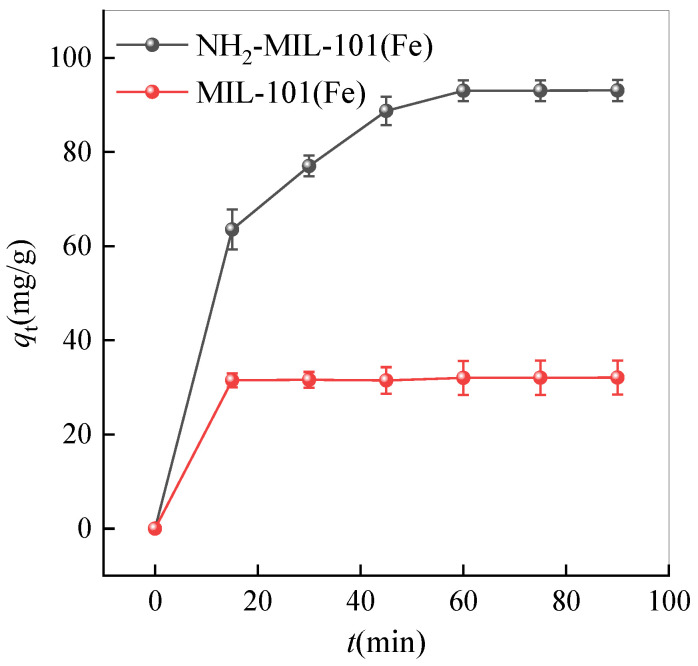
Comparison of adsorption performance between MIL-101(Fe) and NH_2_-MIL-101(Fe).

The comparative adsorption performance of MIL-101(Fe) and NH_2_-MIL-101(Fe) is depicted in the following Figure 7. The adsorption effect of NH_2_-MIL-101 (Fe) reached 93.05 mg/g after adsorption for 90 min, while the adsorption effect of MIL-101(Fe) was only 32.055 mg/g. NH_2_-MIL-101(Fe) demonstrates a 40% improvement in adsorption efficiency compared to MIL-101(Fe) after amino functionalization. This is mainly because the presence of amino groups provides additional adsorption sites for tetracycline molecules during adsorption [52]. Additionally, the graph clearly shows a significant initial increase in adsorption within the first 10 min, attributed to the abundance of initial adsorption active sites on the material surface. Over time, the rate of adsorption increase gradually diminishes, this being particularly evident after one hour, suggesting the saturation of the adsorption active sites, where most sites are occupied by tetracycline. This indicates a nearing equilibrium state, where the material’s effectiveness in adsorbing tetracycline is significantly reduced. Compared with biochar (39.22 mg/g) [53], layered porous ZIF-8 (49.3 mg/g) [54], and polystyrene microspheres (45.0 mg/g) [55], NH_2_-MIL-101 (Fe) has a higher adsorption capacity of TC. Catalyst reuse is crucial for practical applications. The materials for the cyclic experiments were prepared by centrifugation and washing with deionized water and methanol following adsorption. The adsorption efficiency of NH_2_-MIL-101(Fe) decreased from 93.05 mg/g to 80.01 mg/g after five cycles, demonstrating the catalyst’s high stability.

This study employed two widely used kinetic models, pseudo-first-order and pseudo-second-order kinetic models, to fit and systematically analyze the kinetic data during the adsorption process. This step aims to unveil the essence of the adsorption process through mathematical modeling, providing robust theoretical support for understanding the adsorption mechanism.

#### 2.2.1. Pseudo-First-Order Kinetic Model

The critical parameters of the pseudo-first-order kinetic model were obtained through the meticulous handling of equilibrium curve data, as shown in Table 2. The corresponding equation was fitted by plotting ln(*q*_e_ − *q*_t_) against adsorption time t. Subsequently, values of *K*_1_ and *q*_1_ were computed using the slope and intercept of the equation.

Figure S3 illustrates the fitting using the pseudo-first-order kinetic model, revealing noticeable deviations between the actual data and the fitted line. This indicates that the linear fit was not ideal. Moreover, the relatively low coefficient of determination (*R*^2^) in the table further confirms that the pseudo-first-order kinetic model may not be suitable for describing the adsorption of tetracycline on the material.

#### 2.2.2. Pseudo-Second-Order Kinetic Model

Key parameters of the pseudo-second-order kinetic model were calculated and fitted as shown in Table 3. The equation was fitted by plotting t/*q*_t_ against adsorption time t, and values of *K*_2_ and *q*_2_ were computed using the slope and intercept of the equation.

Figure S4 presents the processed kinetic data and fitting using the pseudo-second-order kinetic model, showing a strong linear relationship between the experimental data and the fitted line. Additionally, examination of the data in the table reveals a high coefficient of determination (*R*^2^ = 0.9834) and close agreement between experimental and theoretical values. Based on these analyses, it can be concluded that NH_2_-MIL-101(Fe) conforms well to the adsorption process of tetracycline under the pseudo-second-order kinetic model, indicating that the dominant mechanism of adsorption is chemisorption.

### 2.3. Adsorption Isotherm Analysis

To gain deeper insights into the adsorption mechanisms of the material, this study employed two classical isotherm models, namely the Langmuir and Freundlich models, to fit the adsorption isotherms at temperatures of 15 °C, 25 °C, and 35 °C.

#### 2.3.1. Adsorption Isotherms

Three sets of 100 mL solutions of tetracycline at initial concentrations of 5 mg/L, 10 mg/L, 20 mg/L, 30 mg/L, and 40 mg/L were prepared in conical flasks. Each solution contained 30 mg of NH_2_-MIL-101(Fe) adsorbent and was placed in a temperature-controlled shaker at 15 °C, 25 °C, and 35 °C for 1 h to achieve adsorption equilibrium. Subsequently, the solutions were transferred to colorimetric dishes, and their absorbance was measured using a UV/Vis spectrophotometer. The equilibrium concentrations (*C*_e_) and adsorption capacities (*q*_e_) were calculated based on a standard tetracycline calibration curve.

**Figure 8:**
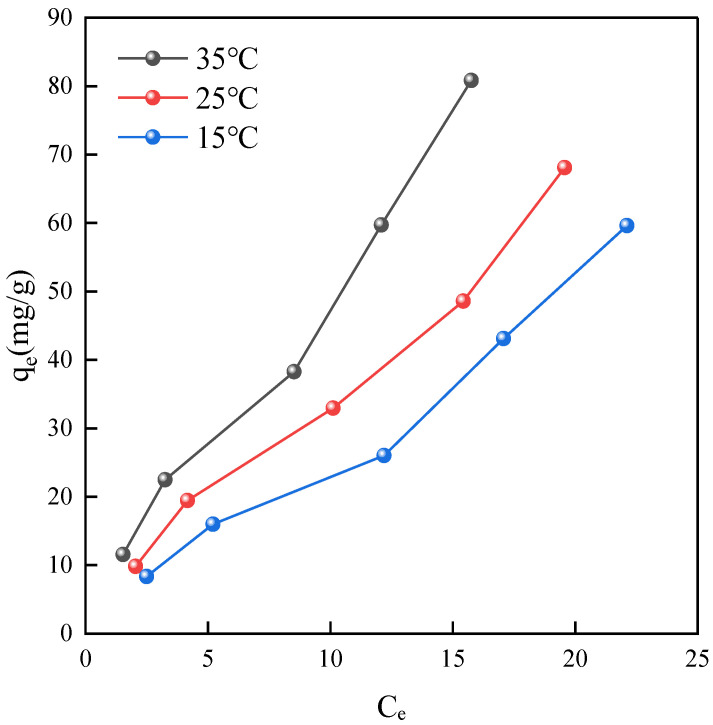
Adsorption isotherms.

When plotting the adsorption isotherms, the equilibrium concentration (*C*_e_) was plotted on the x-axis and the equilibrium adsorption capacity (*q*_e_) on the y-axis. As shown in Figure 8, an increase in equilibrium concentration corresponded to a proportional increase in equilibrium adsorption capacity. Additionally, it was observed that with rising temperatures, the adsorption capacity significantly increased, indicating the material’s pronounced endothermic adsorption characteristics and the temperature-dependent enhancement of adsorption capacity.

#### 2.3.2. Langmuir Model Adsorption Isotherm Fitting

**Figure 9:**
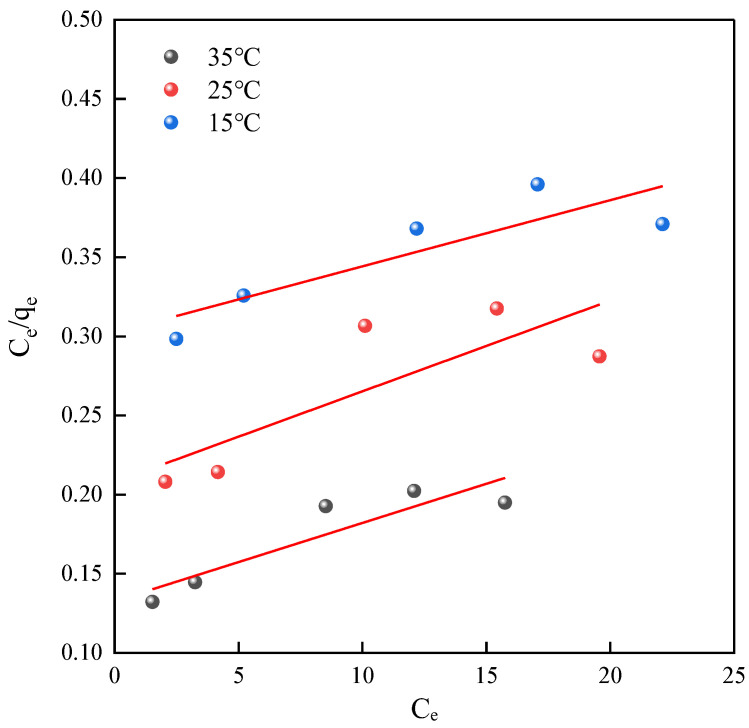
Langmuir Model adsorption isotherm fitting.

For the Langmuir model adsorption isotherm plots, the equilibrium concentration of tetracycline (*Ce*) was plotted against the ratio of *Ce* to *qe* at adsorption equilibrium, as shown in Figure 9. Subsequently, a linear regression analysis was performed on these data, and the resulting fitting equations and related parameters are summarized in Table 4.

From the data in Table 4, it can be inferred that the Langmuir model did not adequately simulate the adsorption of tetracycline on NH_2_-MIL-101(Fe). Hence, it can be concluded that this model is not suitable for elucidating the intrinsic adsorption mechanisms of the material.

#### 2.3.3. Freundlich Model Adsorption Isotherm Fitting

**Figure 10:**
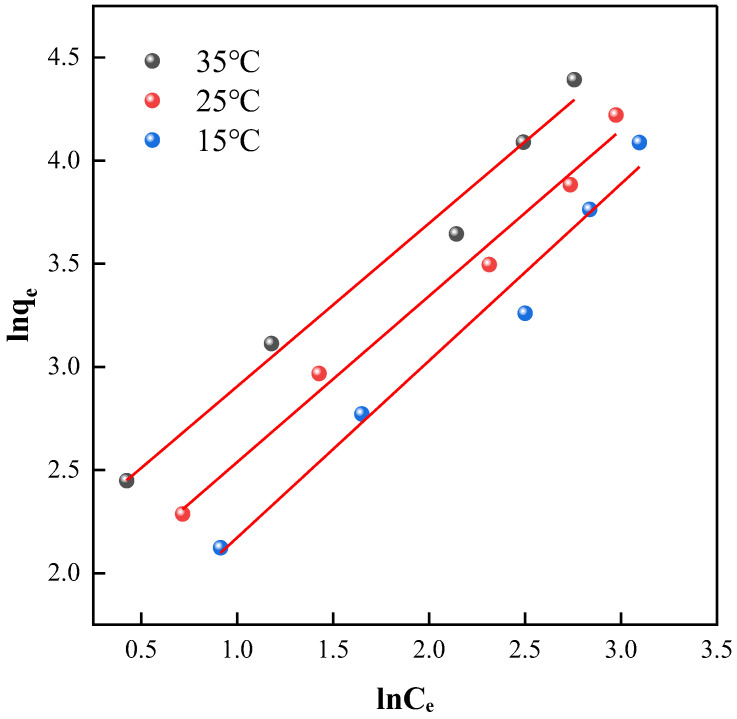
Freundlich Model adsorption isotherm fitting.

For the Freundlich model adsorption isotherm plots, ln*C*_e_ was plotted against lnq*_e_* at each of the three different temperatures, as shown in Figure 10. After fitting these adsorption isotherms, corresponding parameters were obtained and are detailed in Table 5.

Observing the results shown in Figure 10, the Freundlich model demonstrated good applicability in simulating the adsorption isotherms. As indicated in Table 5, the values of n all exceeded 1, reflecting relatively favorable adsorption processes. Moreover, the adsorption rate constants increased with temperature, indicating an increase in adsorption capacity. Furthermore, the linear regression coefficients at all temperatures remained above 0.96, significantly better than those obtained with the Langmuir model. Therefore, for the adsorption of tetracycline on this material, the Freundlich model provides a more suitable explanation, suggesting a heterogeneous adsorption mechanism involving multilayer adsorption and highlighting the complexity of the adsorbent’s surface structure and the diversity of adsorption sites.

### 2.4. Thermodynamics

**Figure 11:**
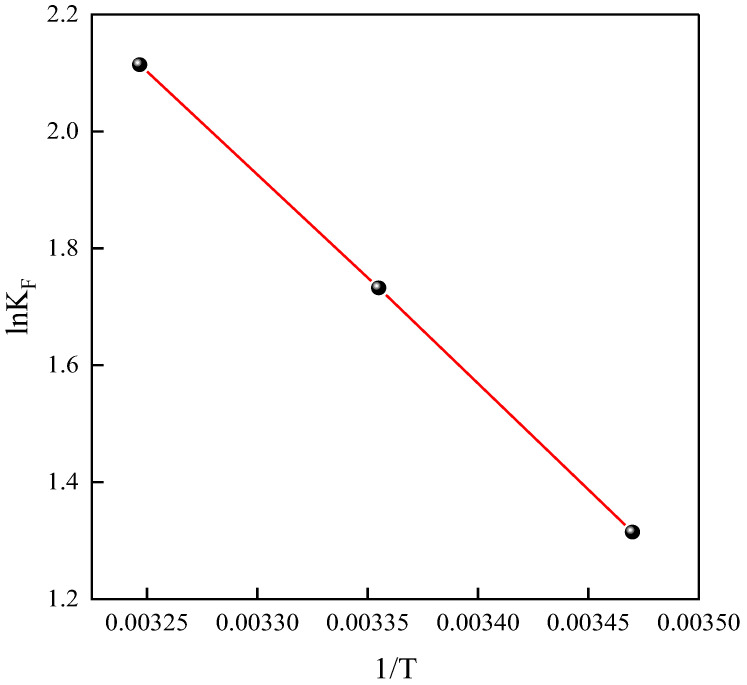
Relationship between ln*K*_F_ and 1/T.

Based on the adsorption isotherm data, the parameters of the Freundlich model were selected for thermodynamic analysis. A linear relationship was observed by plotting ln*K*_F_ against 1/T (temperature). Subsequently, using the slope and intercept of the fitted line, the enthalpy change (∆*H*) and entropy change (∆*S*) during the adsorption process were calculated. Finally, based on these thermodynamic parameters, the Gibbs free energy change (∆*G*) was computed under different temperature conditions, comprehensively analyzing the thermodynamic characteristics of the adsorption process. An examination of Figure 11 reveals a significant linear relationship between ln*K*_F_ and 1/T. Using the slope and intercept of this linear relationship, ∆*H*, ∆*S*, and ∆*G* were calculated and are summarized in Table 6.

From Table 6, it is evident that ∆*G* is less than 0, confirming the spontaneity of the material’s adsorption process for tetracycline. Conversely, the positive ∆*H* value during this adsorption process indicates an endothermic reaction, contradicting the exothermic viewpoint traditionally held in adsorption theory. According to the existing literature, this phenomenon can be reasonably explained by the solvent displacement theory. Specifically, in liquid-phase adsorption, hydrophilic groups on the material surface form hydrogen bonds with water molecules. When tetracycline molecules adsorb, they must overcome these hydrogen bond interactions, leading to the desorption of water molecules. The heat absorbed during the desorption of water molecules exceeds the heat released when tetracycline molecules adsorb, thus demonstrating the endothermic nature of the entire adsorption process.

Moreover, a positive ∆*S* value indicates an overall increase in disorder during the adsorption process, reflecting an increase in system entropy due to the combined effects of desorption and adsorption.

### 2.5. Effect of Material Dosage on Adsorption Efficiency

**Figure 12:**
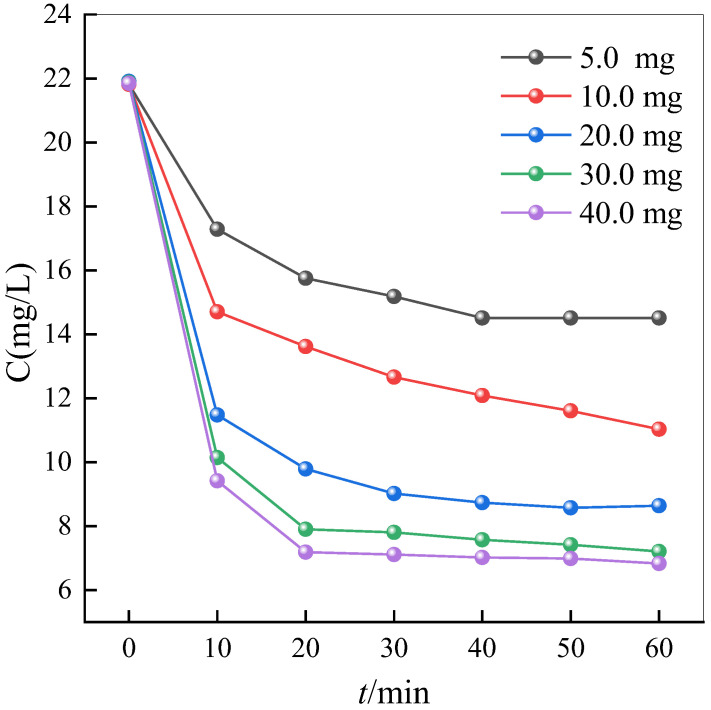
Adsorption efficiency of different NH_2_-MIL-101(Fe) dosages.

Following material preparation, NH_2_-MIL-101(Fe) was respectively dosed at 5, 10, 20, 30, and 40 mg for 100 mL of tetracycline (20 mg/L) to conduct 1 h adsorption studies at room temperature. As depicted in Figure 12, increased dosages of the adsorbent correspond to improved adsorption efficiency.

### 2.6. Effect of pH on Adsorption Capacity

**Figure 13:**
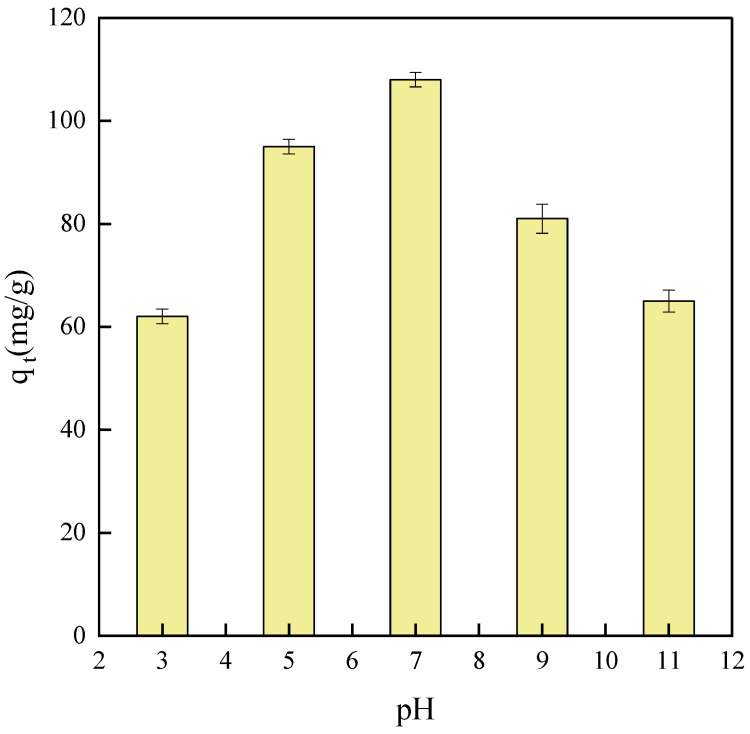
pH effect on NH_2_-MIL-101(Fe) adsorption capacity.

In liquid-phase adsorption, the pH value is a critical parameter significantly influencing the adsorption behavior of materials. Tetracycline exhibits pronounced instability under extreme pH conditions, i.e., strong acid or alkali environments. This instability arises from changes in the molecule’s charge distribution and chemical structure induced by strong acids or bases. Specifically, in strong acid environments, the protonation of the amino groups in tetracycline reduces its solubility and stability. Conversely, under strong alkaline conditions, hydrolysis reactions may occur at the ester and amide bonds of tetracycline, further degrading its structure and stability [38]. As shown in Figure 13, the adsorption capacity varies with pH value. Lower adsorption capacities are observed under acidic and alkaline conditions, while optimal adsorption occurs under neutral conditions. The form of tetracycline present under different pH conditions significantly influences its adsorption behavior. Tetracycline exists predominantly as a cation in acidic environments, shifts to an anion in alkaline conditions, and adopts a zwitterionic form in neutral conditions. This variation in form critically affects the adsorption efficiency of the adsorbent. Notably, under neutral conditions, the balanced distribution of positive and negative charges in the zwitterionic form enhances the interaction between tetracycline molecules and the adsorbent surface through favorable electronic attraction. Furthermore, the stable charge state of the adsorbent surface under neutral conditions minimizes its susceptibility to fluctuations in external pH values, thereby maintaining strong interaction forces with tetracycline molecules and enhancing the adsorption efficiency.

## 3. Materials and Methods

### 3.1. Instruments and Experimental Reagents

#### 3.1.1. Instruments

The main instruments used in this experiment include an electronic analytical balance (DHG-9023A, Shanghai Precision Experimental Equipment Co., Ltd., Shanghai, China), high-pressure reactor (A50, A51, A52, A53, Weihai Xingyu Chemical Machinery Co., Ltd., Weihai, Beijing), high-speed centrifuge (HI850, Hunan Xiangyi Laboratory Instrument Development Co., Ltd., Changsha, China), magnetic stirrer (85-2C, Gongyi Yuhua Instrument Co., Ltd., Gongyi, China), electric constant-temperature drying oven (202, Beijing Yongguangming Medical Instrument Co., Ltd., Beijing, China), UV/Vis spectrophotometer (UV-2700i, Shimadzu Instrument Co., Ltd., Tokyo, Japan), Fourier-transform infrared spectrometer (Nicolet iS50, Thermo Fisher Scientific, Inc., Waltham, MA, USA), X-ray diffractometer (MiniFlex600, Thermo Fisher Scientific, Inc., Waltham, MA, USA), X-ray photoelectron spectrometer (Escalab250Xi, Thermo Fisher Scientific, Inc., Waltham, MA, USA), scanning electron microscope (JSM-7610 FPlus, JEOL Ltd., Tokyo, Japan), specific surface area and pore size analyzer (JWGB JY-BK112, Beijing Jingwei Gaobo Science and Technology Co., Ltd., Beijing, China), thermogravimetric analyzer (STA449-F3, Germany Naichi Instrument Manufacturing Co., Ltd., Dongguang, China), and electronic analytical balance (PHS-3E, Shanghai Yidian Scientific Instrument Co., Ltd., Shanghai, China).

#### 3.1.2. Experimental Reagents

2-aminoterephthalic acid (C_8_H_7_NO_4_, 99%) was purchased from Alfa Aesar (China) Chemical Co., Ltd., Shanghai, China. Ferric chloride hexahydrate (FeCl_3_·6H_2_O, 99%) was purchased from Shanghai McLean Biochemical Technology Co., Ltd., Shanghai, China. Tetracycline hydrochloride (C_22_H_24_N_2_O_8_·HCl, 98%) was purchased from Aladdin Reagent (Shanghai) Co., Ltd., Shanghai, China. Ethanol (C_2_H_6_O, Analytical Pure), methanol (CH_3_OH, Analytical Pure), terephthalic acid (C_8_H_6_O_4_, 99%), and N. N-dimethylformamide (HCON(CH_3_)_2_, 99.5%) were purchased from China National Pharmaceutical Group Chemical Reagent Co., Ltd., Shanghai, China.

### 3.2. Experimental Methods

#### 3.2.1. Material Preparation

Two kinds of MOF materials, MIL-101(Fe) and NH_2_-MIL-101(Fe), were synthesized by a straightforward solvothermal method with reference to previous studies [43,56,57]. In a hydrothermal synthesis reactor, a mixture of FeCl_3_·6H_2_O, terephthalic acid (H_2_BDC) and N, N-dimethylformamide (DMF) was heated at 110 °C for 20 h. After three washes with ethanol and DMF, followed by vacuum activation at 60 °C for 8 h, a reddish-brown MIL-101(Fe) sample was obtained. Similarly, a mixture of FeCl_3_·6H_2_O, 2-aminoterephthalic acid, and DMF underwent reaction in the hydrothermal synthesis reactor at 110 °C for 24 h. The precipitate was washed with DMF and methanol, then left overnight at 60 °C to yield a deep red NH_2_-MIL-101(Fe) sample. Unless otherwise specified, all reagents were dissolved in deionized water at room temperature.

#### 3.2.2. Calculation of Adsorption Capacity

Using an electronic balance, the prepared MOF materials were accurately weighed and added to a tetracycline solution of known concentration. Temperature (25 °C) and stirring speed were carefully controlled throughout the experiment. Samples were taken at predetermined intervals, filtered, and transferred to cuvettes for spectrophotometric analysis. The absorbance of the tetracycline samples was measured precisely using a UV/Vis spectrophotometer (UV-2700i, Shimadzu Instrument Co., Ltd., Tokyo, Japan) at 356 nm. The concentration of tetracycline in the solution was calculated accurately based on a standard curve (Text S1). The adsorption capacity of the materials was determined precisely using specific calculation formulas as outlined in Equation (1).

(1) $$q_{t}=\frac{C_{0}-C_{t}}{m}\times V$$

In the formula, *q*_t_—adsorption capacity at time t, (mg/g);

*C*_0_—initial concentration of tetracycline, (mg/L);

*C*_t_—concentration of tetracycline solution at time t, (mg/L);

*m*—mass of adsorbent, (mg);

*V*—volume of tetracycline solution, (mL).

### 3.3. Material Characterization Methods

#### 3.3.1. X-ray Diffraction Analysis (XRD)

The crystalline structure and phase composition of the materials were characterized using a MiniFlex600 X-ray powder diffractometer (Rigaku Co., Ltd., Tokyo, Japan). The experimental conditions included the use of Cu Kα radiation as the incident source, with a working current of 40 mA and the voltage set to 40 kV. The scanning speed was set at 6°/min over a range of 5° to 90°.

#### 3.3.2. Fourier-Transform Infrared Spectroscopy (FT-IR)

Fourier-transform infrared spectroscopy (FT-IR) involves measuring the absorption and transmission of infrared light by materials to generate characteristic spectra, revealing chemical bonds and functional group information within the material molecules. In this study, a Nicolet iS50 FT-IR spectrometer was employed to investigate the chemical composition, functional groups, and molecular interactions of the synthesized materials. Spectral data analysis was performed using the KBr pellet method within the wavelength range of 400 to 4000 cm^−1^. The number of scans was 32.

#### 3.3.3. Scanning Electron Microscopy

The surface morphology, feature distribution, and particle size of the synthesized materials were analyzed using a JSM-7610 FPlus scanning electron microscope (JEOL Co., Ltd., Tokyo, Japan). The working voltage was set to 10 kV, and a gold spraying treatment (Platinum) was carried out. The working distance was 8.1 mm.

#### 3.3.4. Energy-Dispersive Spectroscopy (EDS)

The elemental distribution of the samples was determined using a scanning electron microscope (JSM-7610 FPlus) equipped with energy-dispersive X-ray spectroscopy.

#### 3.3.5. X-ray Photoelectron Spectroscopy (XPS)

The surface chemical composition and chemical states of the samples were analyzed using an Escalab 250 Xi X-ray photoelectron spectrometer (Thermo Fisher Scientific, Inc., Waltham, MA, USA), calibrated with C 1s = 284.8 eV, to provide insights into the material’s surface properties.

#### 3.3.6. Brunauer–Emmett–Teller Analysis (BET)

A BET analysis is commonly used to characterize the surface properties and pore structures of solid materials, such as porous materials. A JWGB JY-BK 112 specific surface area analyzer was used to test the specific surface area, pore volume, and pore diameter of the sample at 100 °C for 2 h.

#### 3.3.7. Thermogravimetric Analysis (TGA)

Thermogravimetric analysis (TGA) is a thermal analysis technique used to measure the mass change of a substance as a function of temperature during heating or cooling. The STA449-F3 thermal analyzer was utilized to assess the thermal stability of the samples. The experimental temperature range was 50 °C–700 °C, the heating rate was 10 °C/min, and the test atmosphere was nitrogen.

### 3.4. Analysis of Adsorption Mechanisms

#### 3.4.1. Adsorption Kinetics Models

Two different kinetic models were applied, a pseudo-first-order kinetic model and pseudo-second-order kinetic model, to linearly fit the experimental adsorption data, accurately determining the adsorption mechanism of the materials.

##### Pseudo-First-Order Kinetic Model

If the kinetic data fitting tends towards a pseudo-first-order kinetic adsorption model, this trend indicates that the dominant mechanism governing the adsorption process is diffusion. The specific linear equation is expressed as shown in Equation (2):

(2) $$ln\left(q_{e}-q_{t}\right)=lnq_{1}-K_{1}t$$

In the formula, *t*—adsorption time, min;

*q*_t_—adsorption capacity at time t, mg/g;

*q*_e_—adsorption capacity at adsorption equilibrium, mg/g;

*q*_1_—theoretical adsorption capacity calculated after fitting, mg/g;

*K*_1_—pseudo-first-order kinetic constant, min^−1^.

##### Proposed Second-Order Kinetic Model

If the kinetic data conform to the pseudo-second-order kinetic adsorption model, it indicates that the adsorption behavior is mainly controlled by the chemical adsorption mechanism. The linear equation is shown in Equation (3):

(3) $$\frac{t}{q_{t}}=\frac{1}{K_{2}{q_{2}}^{2}}+\frac{t}{q_{2}}$$

In the formula, *q*_t_—adsorption capacity at time t, mg/g;

*q*_e_—adsorption capacity at adsorption equilibrium, mg/g;

*q*_2_—theoretical adsorption capacity calculated after fitting, mg/g;

*K*_2_—pseudo-second-order kinetic constant, g/(mg·min).

#### 3.4.2. Adsorption Isotherm Model

In this study, two widely recognized adsorption models, namely the Langmuir adsorption isotherm model and Freundlich adsorption isotherm model, were used for in-depth simulation analysis.

##### Langmuir Adsorption Isotherm Model

The linear equation of the model is shown in Equation (4):

(4) $$\frac{C_{e}}{q_{e}}=\frac{1}{K_{L}q_{L}}+\frac{C_{e}}{q_{L}}$$

In the formula, *q*e—adsorption capacity at adsorption equilibrium, mg/g;

*q*_L_—maximum adsorption capacity of the material calculated after fitting, mg/g;

*C*_e_—concentration of tetracycline at adsorption equilibrium, mg/L;

*K*_L_—Langmuir adsorption constant, L/mol.

The adsorption constant is a key parameter for measuring the difficulty of adsorption reactions [3]. Specifically, when the *K*_L_ value is 0, the reaction is irreversible; when the *K*_L_ value is between 0 and 1, the reaction can occur; when the *K*_L_ value is exactly 1, it represents linear adsorption; however, once the *K*_L_ value exceeds 1, the reaction has more difficulty in proceeding.

##### Freundlich Adsorption Isotherm Model

The linear equation of this model is shown in Equation (5):

(5) $$lnq_{e}=\frac{1}{n}lnC_{e}+lnK_{F}$$

In the formula, *q*_e_—adsorption capacity at adsorption equilibrium, mg/g;

*C*_e_—concentration of tetracycline at adsorption equilibrium, mg/L;

*K*_F_—Freundlich adsorption constant, L/mol;

*n*—Freundlich nonlinear exponent.

The size of the *K*_F_ value directly reflects the adsorption capacity of the adsorbent; that is, the larger the *K*_F_ value, the stronger the adsorption capacity of the adsorbent. On the contrary, the smaller the *K*_F_ value, the weaker the adsorption capacity of the adsorbent. The *n* value is used to describe the difficulty level of the adsorption process. When the *n* value is greater than 1, it indicates that the adsorption process has relative ease in proceeding.

#### 3.4.3. Adsorption Thermodynamics

To delve into the thermodynamic properties of the adsorption process, this study aims to elucidate the spontaneity and thermal effects of the adsorption reaction. Therefore, it is crucial to perform thermodynamic calculations of the adsorption process. Enthalpy change (∆*H*) is a critical parameter that directly reflects the heat effects of the adsorption process. Entropy change (∆*S*) describes the change in system disorder or randomness. The Gibbs free energy change (∆*G*) is a key thermodynamic parameter that assesses the spontaneity of a chemical reaction, determining whether the adsorption reaction can proceed spontaneously. In this study, the adsorption process was best fitted with the Freundlich model; thus, the Freundlich model was selected for calculating the relevant thermodynamic parameters: enthalpy change (∆*H*), entropy change (∆*S*), and Gibbs free energy change (∆*G*).

The enthalpy change (∆*H*) and entropy change (∆*S*) were calculated using the van ’t Hoff equation:

(6) $$lnK_{F}=\;-\frac{∆H}{8.314T}+\frac{∆S}{8.314}$$

The equation for calculating ∆*G* is as follows:

(7) $$∆G=\;-8.314TlnK_{F}$$

## 4. Conclusions

NH_2_-MIL-101(Fe) materials were successfully synthesized via a solvothermal method, and their synthesis was confirmed through various characterization techniques including X-ray diffraction (XRD), Fourier-transform infrared spectroscopy (FT-IR), scanning electron microscopy, energy-dispersive X-ray spectroscopy (EDS), and X-ray photoelectron spectroscopy (XPS). NH_2_-MIL-101(Fe) exhibits a stable crystalline morphology, uniform particle size, smooth surface, and typical octahedral structure. BET analysis revealed a slight decrease in the specific surface area and pore size of NH_2_-MIL-101(Fe) compared to MIL-101(Fe), attributed to the introduction of amino groups. Additionally, thermogravimetric analysis (TGA) demonstrated the excellent thermal stability of NH_2_-MIL-101(Fe). The adsorption kinetics of tetracycline onto NH_2_-MIL-101(Fe) followed a pseudo-second-order kinetic model, suggesting the dominance of chemisorption in the adsorption process. Isothermal adsorption studies indicated a good fit with the Freundlich model, highlighting multilayer molecular adsorption characteristics. Furthermore, thermodynamic data indicated that the adsorption of tetracycline onto NH_2_-MIL-101(Fe) is an endothermic and spontaneous process, suggesting enhanced adsorption efficiency with increased temperatures. Investigation into the effects of adsorbent dosage, temperature, and pH on the adsorption performance of NH_2_-MIL-101(Fe) towards tetracycline revealed that a higher adsorbent dosage and temperature positively influence adsorption efficiency. Conversely, extremes of pH (both acidic and alkaline) led to a decreased adsorption capacity, with optimal performance observed under neutral conditions. This study provides technical support for the removal of tetracycline antibiotics from aqueous environments through the adsorption processes facilitated by NH_2_-MIL-101(Fe).

## Figures and Tables

**Table 1:** Specific surface area and average pore diameter data for MIL-101(Fe) and NH_2_-MIL-101(Fe).

Material	Specific Surface Area (m^2^/g)	Average Pore Size (nm)
NH_2_-MIL-101(Fe)	403.55	2.43
MIL-101(Fe)	1236.71	2.75

**Table 2:** Key parameters of the pseudo-first-order kinetic model.

Material	Pseudo-First-Order Kinetic Model			
	*q_e_*	*q*_1_	*K*_1_	*R* ^2^
NH_2_-MIL-101(Fe)	107.815	102.3604	0.1533	0.918

**Table 3:** Key parameters of the pseudo-second-order kinetic model.

Material	Pseudo-Second-Order Kinetic Model			
	*q_e_*	*q*_2_	*K*_2_	*R* ^2^
NH_2_-MIL-101(Fe)	107.8150	110.3752	0.0021	0.9834

**Table 4:** Langmuir Model adsorption isotherm parameters.

Material	*T*/°C	Langmuir Equation	*q_m_*	*K_L_*	*R* ^2^
NH_2_-MIL-101(Fe)	15	*y* = 0.0041*x* + 0.3024	244	0.0136	0.6736
	25	*y* = 0.0057*x* + 0.2079	175	0.0275	0.5515
	35	*y* = 0.0050*x* + 0.1326	200	0.03771	0.7637

**Table 5:** Freundlich Model adsorption isotherm parameters.

Material	*T*/°C	Freundlich Equation	*n*	*K_F_*	*R* ^2^
NH_2_-MIL-101(Fe)	15	*y* = 0.8578*x* + 1.3145	1.1658	3.7229	0.9694
	25	*y* = 0.8057*x* + 1.7323	1.2412	5.6536	0.9832
	35	*y* = 0.7914*x* + 2.1139	1.2636	8.2805	0.9774

**Table 6:** Thermodynamic data.

Material	*T/K*	∆*G* (kJ/mol)	∆*H* (kJ/mol)	∆*S* (J/mol/K)
NH_2_-MIL-101(Fe)	288	−3.15	29.38	112.97
	298	−4.29		
	308	−5.41		

## Data Availability

The original contributions presented in the study are included in the article/Supplementary Materials, further inquiries can be directed to the corresponding author.

## Supplementary Materials

The following supporting information can be downloaded at: https://www.mdpi.com/article/10.3390/ijms25189855/s1.
