# Supplementary material for: Preparation of NH2-MIL-101(Fe) Metal Organic Framework and Its Performance in Adsorbing and Removing Tetracycline
Source: Int J Mol Sci. 2024 Sep 12;25(18):9855. doi: 10.3390/ijms25189855 (PMC11432026; doi:10.3390/ijms25189855)
Supplement: Supplementary file 1 [file ijms-25-09855-s001.zip › ijms-3129691-supplementary.pdf]

Article

Preparation of NH_2-MIL-101 (Fe) metal organic framework and its performance in adsorbing and removing tetracycline

Yiting Luo[1,2], Rongkui Su[3,4,*]

[1] Hunan First Normal University, Changsha 410114, China; yitingL2021@163.com (Y.L.)

[2] National Engineering Laboratory of Southern Forestry Ecological Application Technology, Changsha 410004, China; yitingL2021@163.com (Y.L.)

[3] PowerChina Zhongnan Engineering Corporation Limited, Changsha 410004, China; sususufree@163.com (R.S.)

[4] College of Life and Environmental Sciences, Central South University of Forestry and Technology, Changsha, 410004, PR China; sususufree@163.com (R.S.)

* Correspondence: sususufree@163.com (R.S.);

*To whom correspondence should be addressed. Phone: 86-731-85623372; fax: +86-731-85623372; Rongkui Su. e-mail address: sususufree@163.com;

List of Contents

Text S1 Standard curve for tetracycline concentration determination R1

Figure S1 Standard curve of tetracycline solution in deionized water

Figure S2 SEM image of the MIL-101(Fe) R2

Figure S3 Fitting diagram of the first-order kinetic model R3

Figure S4 Fitting diagram of pseudo second order dynamic model R3

Text S1 Standard curve for tetracycline concentration determination

Standard tetracycline solutions in deionized water with concentrations of 2 mg/L, 4 mg/L, 6 mg/L, 8 mg/L and 10 mg/L were configured at room temperature . pH not adjusted. The absorbance value of each standard solution was measured by ultraviolet spectrophotometer at the wavelength of 356nm. The absorbance value of each standard solution was taken as the horizontal coordinate and the concentration of tetracycline solution was taken as the vertical coordinate. The linear equation is y=31.8931x+0.1891 (R^2=0.9998).

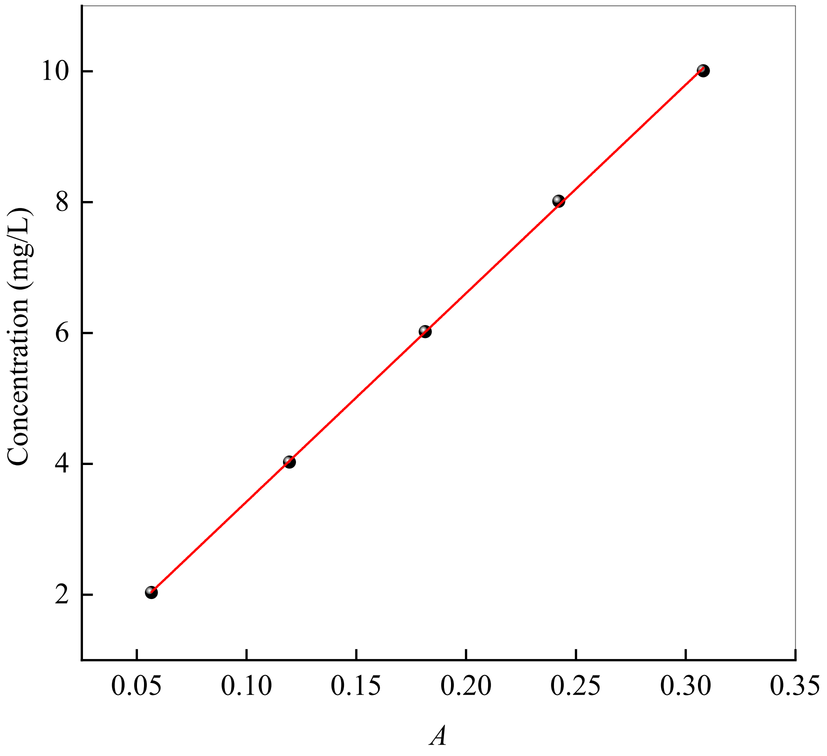

Figure S1 Standard curve of tetracycline solution in deionized water

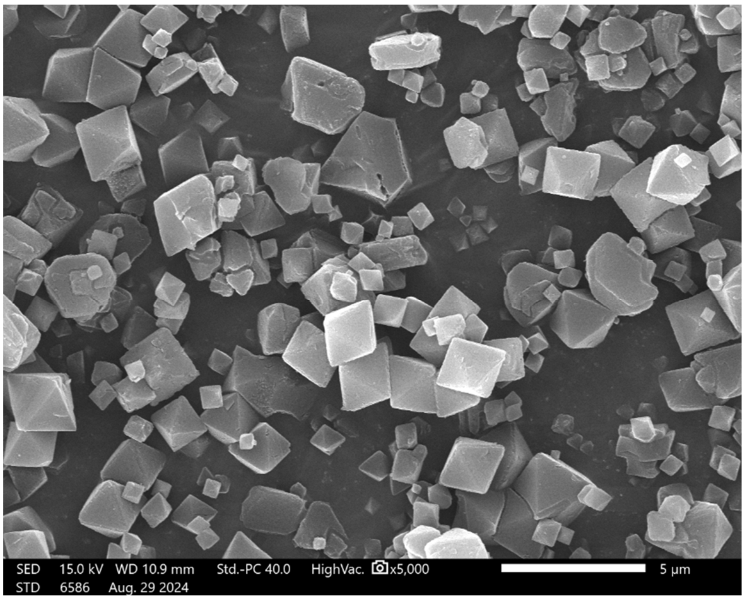

Figure S2 SEM image of the MIL-101(Fe)

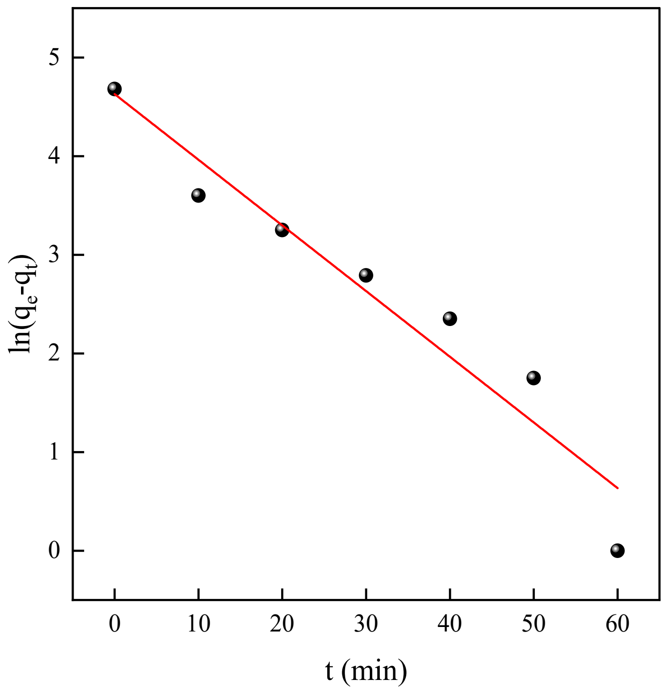

Figure S3 Fitting diagram of the first-order kinetic model

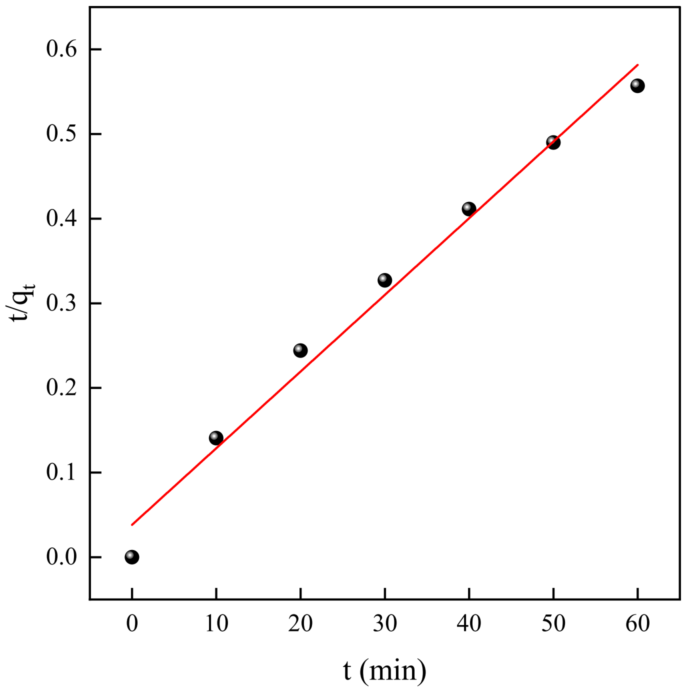

Figure S4 Fitting diagram of pseudo second order dynamic model
